# Supplementary material for: Are Dual-Purpose Chickens Twice as Good? Measuring Performance and Animal Welfare throughout the Fattening Period
Source: Animals (Basel). 2020 Oct 28;10(11):1980. doi: 10.3390/ani10111980 (PMC7692664; doi:10.3390/ani10111980)
Supplement: Supplementary file 1 [file animals-10-01980-s001.pdf]

TABLE 1 QUALITATIVE BEHAVIOR ASSESSMENT

Table S1: Presented are the mean values of the Area under curve (AUC) per term of the Qualitative Behavior Assessment over the whole assessment period of 20 weeks. Means ± SE are given per genotype (Lohmann Brown LB, Lohmann Dual LD, Rhinelander RL), including statistics. The highest possible value would be 4.828 based on calculations with minimum vs. maximum VAS value. For details, see the Methodology section.

		**Mean ± SE**		***F***	**η^2**	**P**	**Post-Hoc**
	**LB**	**LD**	**RL**	**(2;51)**	**N = 18**	**α = 0.05* 0.01** 0.001*** **	
Active	4.40 ± 0.05	4.19 ± 0.07	4.11 ± 0.12	3.259	0.113	0.047*	LB – LD = 0.049
Energetic	4.40 ± 0.05	4.13 ± 0.11	4.24 ± 0.11	2.110	0.076	0.132	
Playful	4.27 ± 0.08	4.03 ± 0.07	3.49 ± 0.08	27.648	0.520	≤ 0.001***	LB – RH < 0.001<br>LD – RH < 0.001
Confident	4.64 ± 0.06	4.63 ± 0.06	4.30 ± 0.16	3.483	0.120	0.038*	
Content	0.44 ± 0.07	4.33 ± 0.10	4.41 ± 0.07	0.408	0.016	0.667	
Friendly	4.61 ± 0.05	4.59 ± 0.04	4.39 ± 0.08	3.807	0.130	0.029*	LB – RH = 0.048
Positively Occupied	4.67 ± 0.04	4.65 ± 0.05	4.51 ± 0.06	3.061	0.107	0.056	
Calm	3.61 ± 0.13	3.96 ± 0.10	3.82 ± 0.07	3.104	0.109	0.053	
Comfortable	4.27 ± 0.06	4.10 ± 0.09	4.32 ± 0.05	2.659	0.094	0.080	
Drowsy	3.18 ± 0.18	3.49 ± 0.15	3.62 ± 0.17	1.827	0.067	0.171	
Relaxed	4.08 ± 0.07	4.25 ± 0.07	4.13 ± 0.06	1.611	0.059	0.210	
Bored	3.06 ± 0.40	3.57 ± 0.26	2.68 ± 0.25	2.044	0.074	0.140	
Depressed	0.17 ± 0.17	0.52 ± 0.28	0.35 ± 0.24	0.555	0.021	0.578	
Frustrated	1.03 ± 0.29	1.21 ± 0.34	0.32 ± 0.22	2.694	0.095	0.077	
Tense	0.86 ± 0.30	1.49 ± 0.27	1.72 ± 0.31	2.297	0.083	0.111	
Agitated	1.16 ± 0.28	1.43 ± 0.30	2.03 ± 0.33	2.166	0.078	0.125	
Helpless	0.33 ± 0.18	0.24 ± 0.17	0.29 ± 0.20	0.060	0.002	0.942	
Nervous	0.54 ± 0.22	0.36 ± 0.20	0.69 ± 0.29	0.498	0.019	0.610	
Unsure	0.42 ± 0.24	0.29 ± 0.20	1.34 ± 0.35	4.492	0.150	0.016*	LD – RH = 0.024
Distressed	0.26 ± 0.18	0.33 ± 0.23	0.80 ± 0.32	1.381	0.051	0.260	
Fearful	1.60 ± 0.27	2.06 ± 0.20	3.59 ± 0.11	0.511	26.655	≤ 0.001***	LB – RH < 0.001<br>LD – RH < 0.001
Scared	0.13 ± 0.13	0.29 ± 0.20	0.47 ± 0.33	0.540	0.021	0.586	

TABLE 2 WEIGHTS

Table S2: Averaged weights [g] for cocks per genotype and week of life (mean ± SE) throughout the fattening period. Exceeding of the 2-kg limit in live weight is marked.

Week of life	Lohmann Dual	Lohmann Brown	Rhinelander
Hatching	43 ± 0.6	39 ± 0.6	39.0 ± 1.0
1	121 ± 1.8	81 ± 1.2	78.0 ± 4.8
2	251 ± 4.3	154 ± 2.0	126 ± 10.5
3	438 ± 7.6	260 ± 3.3	202 ± 7.0
4	675 ± 13.2	396 ± 5.5	316 ± 10.5
5	949 ± 13.8	522 ± 8.3	427 ± 14.9
6	1,243 ± 18.4	707 ± 8.4	604 ± 24.5
7	1,576 ± 26.1	906 ± 10.7	747 ± 30.0
8	1,905 ± 34.7	1,106 ± 13.3	931 ± 28.4
9	2,242 ± 33.0	1,320 ± 16.0	1,125 ± 34.1
10	2,565 ± 41.4	1,517 ± 28.8	1,307 ± 41.2
11	2,894 ± 60.4	1,769 ± 26.1	1,522 ± 49.3
12	3,150 ± 47.7	1,975 ± 27.6	1,620 ± 43.4
13	3,333 ± 49.7	2,027 ± 28.0	1,818 ± 59.7
14	3,454 ± 47.4	2,192 ± 30.0	1,904 ± 62.9
15	3,651 ± 60.9	2,297 ± 27.7	2,164 ± 66.2
16	3,734 ± 64.3	2,497 ± 32.3	2,236 ± 80.6
17	3,919 ± 60.9	2,612 ± 31.6	2,299 ± 75.2
18	4,057 ± 60.7	2,741 ± 34.0	2,441 ± 82.7
19	4,063 ± 62.8	2,827 ± 36.6	2,488 ± 81.7
20	4,071 ± 47.9	2,893 ± 29.3	2,454 ± 65.8

TABLE 3 DAILY WEIGHT GAIN

Table S3: Daily weight gain as mean (M) ± standard error (SE) for each experimental group, focal (F) and random (R) weighed animals per genotype and week of life. Stars indicate maximum daily weight gain.

Week of life	Lohmann Dual			Lohmann Brown			Rhinelander		
	F_M ± SE [g]	R_M [g]	LD_M [g]	F_M ± SE [g]	R_M [g]	LB_M [g]	F_M ± SE [g]	R_M [g]	RL_M [g]
1	11.84 ± 0.20	10.58	11.21	6.36 ± 0.14	5.75	6.05	3.94 ± 0.25		1.97
2	19.51 ± 0.40	17.58	18.54	10.19 ± 0.14	10.56	10.37	7.29 ± 0.39	6.48	6.88
3	28.72 ± 0.45	24.79	26.76	14.93 ± 0.24	15.43	15.18	11.52 ± 0.53	10.31	10.92
4	35.40 ± 0.70	32.39	33.89	19.22 ± 0.37	19.54	19.38	14.36 ± 0.52	18.13	16.24
5	38.71 ± 1.08	38.61	38.66	17.72 ± 0.46	18.33	18.02	16.26 ± 0.79	15.47	15.86
6	42.97 ± 1.42	41.65	42.31	26.70 ± 0.53	26.24	26.47	21.67 ± 0.97	28.73	25.20
7	52.55 ± 1.48	42.56	47.55	28.33 ± 0.50	28.46	28.39	23.69 ± 1.25	17.30	20.50
8	46.15 ± 3.90	48.06	47.11	29.97 ± 0.50	27.18	28.57	27.83 ± 2.85	24.70	26.26
9	50.11 ± 3.87	46.01	48.06*	30.11 ± 0.84	30.96	30.53	24.49 ± 3.38	30.99	27.74
10	48.64 ± 2.29	41.47	45.05	27.28 ± 2.00	28.96	28.12	28.00 ± 1.29	22.84	25.42
11	52.89 ± 3.05	40.90	46.90	36.96 ± 3.80	33.40	35.18*	24.40 ± 1.45	38.44	31.42
12	33.36 ± 2.24	39.89	36.63	30.36 ± 1.20	28.67	29.51	19.96 ± 1.93	7.90	13.93
13	26.49 ± 3.02	25.83	26.16	3.75 ± 1.30	10.93	7.34	26.06 ± 1.47	30.67	28.37
14	16.29 ± 1.49	18.23	17.26	26.46 ± 1.01	20.88	23.67	20.44 ± 2.16	3.97	12.21
15	29.32 ± 1.84	27.06	28.19	11.58 ± 1.16	18.24	14.91	29.23 ± 4.44	45.02	37.13*
16	16.56 ± 2.21	7.20	11.88	29.79 ± 1.57	27.52	28.65	10.17 ± 2.81	10.68	10.43
17	26.32 ± 2.12	26.48	26.40	20.48 ± 0.92	12.34	16.41	11.83 ± 2.91	4.80	8.31
18	15.05 ± 2.25	24.45	19.75	14.58 ± 1.71	22.12	18.35	19.01 ± 1.80	18.93	18.97
19	2.49 ± 1.96	-0.95	0.77	15.96 ± 1.45	8.71	12.33	6.57 ± 1.46	6.71	6.64
20	-0.40 ± 1.58	2.79	1.19	7.09 ± 1.85	11.69	9.39	4.22 ± 3.90	-8.18	-1.98
M	29.65 ± 1.88	27.78	28.71	20.39 ± 1.08	20.29	20.34	17.55 ± 1.83	17.57	17.12

TABLE 4 PRINCIPAL COMPONENT ANALYSIS

Table S4: Loading terms on component 1 and 2 in the principal component analysis.

Terms	Component 1	Loading	Term	Component 2
Depressed	0.902	Positive	Fearful	0.586
Helpless	0.900		Comfortable	0.530
Unsure	0.888			
Scared	0.866			
Distressed	0.865			
Nervous	0.844			
Frustrated	0.748			
Agitated	0.679			
Drowsy	0.670			
Tense	0.620			
Confident	-0.882	Negative	Bored	-0.833
Positively Occupied	-0.770		Playful	-0.361
Content	-0.768		Calm	-0.339
Friendly	-0.757			
Active	-0.705			
Energetic	-0.587			
Relaxed	-0.419			
